# Synergy between Group 2 capsules and lipopolysaccharide underpins serum resistance in extra-intestinal pathogenic Escherichia coli

**DOI:** 10.1099/mic.0.001493

**Published:** 2024-08-23

**Authors:** Naoise McGarry, Domhnall Roe, Stephen G. J. Smith

**Affiliations:** 1 Department of Clinical Microbiology, School of Medicine, Trinity College, Dublin, Republic of Ireland

**Keywords:** antibiotic resistance, capsule, *Escherichia coli*, ExPEC, LPS, polysaccharides, serum resistance

## Abstract

*Escherichia coli (E. coli*) is a major cause of urinary tract infections, bacteraemia, and sepsis. CFT073 is a prototypic, urosepsis isolate of sequence type (ST) 73. This laboratory, among others, has shown that strain CFT073 is resistant to serum, with capsule and other extracellular polysaccharides imparting resistance. The interplay of such polysaccharides remains under-explored. This study has shown that CFT073 mutants deficient in lipopolysaccharide (LPS) O-antigen and capsule display exquisite serum sensitivity. Additionally, O-antigen and LPS outer core mutants displayed significantly decreased surface K2 capsule, coupled with increased unbound K2 capsule being detected in the supernatant. The R1 core and O6 antigen are involved in the tethering of K2 capsule to the CFT073 cell surface, highlighting the importance of the R1 core in serum resistance. The dependence of capsule on LPS was shown to be post-transcriptional and related to changes in cell surface hydrophobicity. Furthermore, immunofluorescence microscopy suggested that the surface pattern of capsule is altered in such LPS core mutants, which display a punctate capsule pattern. Finally, targeting LPS biosynthesis using sub-inhibitory concentrations of a WaaG inhibitor resulted in increased serum sensitivity and decreased capsule in CFT073. Interestingly, the dependency of capsule on LPS has been observed previously in other *Enterobacteria*, indicating that the synergy between these polysaccharides is not just strain, serotype or species-specific but may be conserved across several pathogenic Gram-negative species. Therefore, using WaaG inhibitor derivatives to target LPS is a promising therapeutic strategy to reduce morbidity and mortality by reducing or eliminating surface capsule.

**Impact Statement**

Disseminated infections caused by *E. coli* place a large burden on healthcare systems globally, with incidence as well as antimicrobial resistance on the rise. The key to designing successful therapeutic strategies is to understand how and why bacteria cause infection, with an increased need for alternative/ preventative therapies in the age of antimicrobial resistance. ST131 is the predominant and most significant clonal group of bloodstream isolates currently. The second-most frequently isolated clonal group in *E. coli* bloodstream infections is that of ST73, into which the prototype used in this study, CFT073, is categorised. Whilst antimicrobial resistance is not currently widespread in the ST73 clonal group, patterns of drug resistance are emerging. The clonal group is also associated with higher virulence scores than ST131, highlighting a need to better understand ST73 virulence to identify potential therapeutic intervention strategies. This study has identified a synergy between common virulence factors of sepsis-associated *E. coli*, capsule and LPS, which can be exploited to reduce virulence, a promising prospect in the age of antimicrobial resistance.

## Data Summary

Oligonucleotide primers for mutagenesis as well as schematic representations of gene clusters were designed based on the assembled genome sequence of strain CFT073 (GenBank accession number: AE014075.1).

## Introduction

Extra-intestinal pathogenic *Escherichia coli* (ExPEC) is a collective term for pathogenic strains of *E. coli* which are responsible for extra-intestinal disease, including meningitis, UTIs and bloodstream infection [1]. These strains possess genes encoding specialised virulence factors which enable the bacteria to colonise the extra-intestinal sites, in addition to evasion of the host immune responses [2]. ExPEC are distinct from intestinal pathogenic *E. coli* in terms of the virulence factors and mechanisms of pathogenesis predominant amongst strains of these extra-intestinal pathotypes, this can be attributed to the ability of *E. coli* to adapt to its niche [3].

Mammalian serum displays a wide variety of highly effective innate immune responses to *E. coli* colonisation and bloodstream infection, including the complement system and lysozyme [2]. Thus, in order for ExPEC to successfully colonise the host and survive in the blood, these bacteria encode several virulence factors which confer serum resistance. Extracellular polysaccharide factors such as colanic acid, the LPS component O antigen and the surface-associated polysaccharide capsule (K capsule) comprise the extracellular glycome and have been implicated in the resistance of ExPEC to human serum [2,4,6]. A review previously published by this group explores the bactericidal components of human serum, and how ExPEC overcome these defences, in detail [2].

LPS forms a core part of the Gram-negative cell envelope and is essential for structural integrity as well as virulence, attachment, and adhesion to surfaces [7]. LPS is comprised of three compartments, the highly conserved lipid A, oligosaccharide core and the highly immunogenic O antigen [8]. The LPS O antigen, in juxtaposition to the conserved lipid A, is varied and is used as a serotyping method for *E. coli*. The O antigen is a highly variable region, differing amongst strains and thus, can contribute to pathogenicity. More than 180 different *E. coli* O serotypes have been identified and characterised, with some O antigen types such as O1, O2, O4, O6, O7, O8, O16, O18, O25, and O75 commonly identified in ExPEC and specifically, UTI-associated isolates [5]. The O2, O4, O6, O7 and O25 antigens have been specifically implicated in serum resistance of clinical urosepsis isolates and prototypic strains [5,7, 9].

In contrast to the variable O antigen, there exists only five core types in *E. coli:* K-12, R1, R2, R3 and R4. These core groups are differentiated by their outer core structures [10,11]. The R1-type core is the most frequent amongst *E. coli* strains, present in approximately 68% of sequenced strains [10]. Interestingly, different core types are correlated with distinct pathotypes, as is also seen with certain O types (i.e. O25 association with extra-intestinal infections) [12]. Shiga-toxin producing *E. coli* isolates are predominantly of the R3 core type, whilst many non-pathogenic, commensal isolates are of the K-12 core type [10]. Moreover, the R1 core type strains comprise 100% of phylogenetic group B2 *E. coli*, the phylogroup most associated with extra-intestinal infections, indicating a link between core type and pathogenesis/ site of infection [13].

The exopolysaccharide capsule is a cell surface structure composed of long-chain polysaccharides, encasing many isolates and pathotypes of *E. coli*. This exopolysaccharide is similar to those expressed by *Neisseria meningitidis* and *Haemophilus influenzae* among other virulent pathogens, and in those species, capsule is often associated with disseminated infection, immune evasion, and resistance to phagocytosis [14,16]. Due to variations in the capsule composition and structures, more than 80 serologically unique K antigens exist across all strains of *E. coli* [17]. The *E. coli* capsule fulfils a variety of roles in addition to acting as a steric barrier to the complement system and shielding against other host defences as mentioned above [2]. Additionally, the capsule protects against physical environmental stresses, such as dehydration or desiccation [18]. Moreover, secreted *E. coli* polysaccharide capsules can contribute to and/or inhibit biofilm formation, depending on the specific K serotype [19,20].

Despite the hypervariability, there are only two assembly pathways for *E. coli* capsular polysaccharides, designated as Group 1 and 2 prototypes. Groups 3 and 4 are genetic variants of the Group 2 and 1 systems, respectively [21]. Each capsule group encodes a distinctive set of cytosolic and inner-membrane enzymes, which generate a distinct capsule sugar structure and defines the given K serotype [17]. Following biosynthesis, a multiprotein complex translocates the nascent capsule polysaccharide across the inner membrane to the outer envelope, where the capsule structure is assembled on the cell surface [6]. Most ExPEC express Group 2 capsules on their surfaces with K antigens K1, K2, K5, K100 and K92 being most prevalent amongst ExPEC isolates [20]. Furthermore, the K1, K2, K5 and K92 capsules are not just associated with virulent ExPEC isolates but have been shown to be specifically and significantly implicated in the resistance of the isolates to serum [5,6, 22, 23].

Whilst components of the capsule transport and biosynthesis operons (such as KpsC, KpsS, KpsT) are outer membrane-associated, the mature K capsular polysaccharide is intimately associated with the outer envelope through physiochemical interactions [24]. The specific mechanisms which retain capsular polysaccharides to the cell surface are undefined. However, it is strongly evidenced that K1 and K2 capsules (both Group 2 capsules) interact with surface factors such as Braun’s lipoprotein (K2) and LPS core oligosaccharide (K1) to remain associated with the cell, with mutations in these key envelope components resulting in decreased capsule detected at the cell surface [25,27]. Interestingly, interactions between the outer core moiety of LPS and capsule polysaccharides have been observed previously in several *Klebsiella* strains of different serotypes [28,29], in addition to *E. coli* K1 and K5 [25,27].

The aim of this study was to determine whether the synergy between LPS and capsule may be conserved and exists beyond *Klebsiellae* and *E. coli* K1 and K5, in addition to constructing a mutant deficient in both polysaccharides to determine the full contribution of the glycome to serum resistance in an ExPEC prototype, CFT073. Strain CFT073 is of O6:K2:H1 serotype and thus, proves an appropriate model organism to explore the above aims, as whilst O6 and K2 polysaccharides are among those most commonly associated with ExPEC, these serogroups are less characterised than the well-studied capsules K1 and K5 or O antigens O1 and O18 [30,31].

## Methods

### Bacterial strains and culture conditions

The bacterial strains and plasmids utilised in this study are listed in Tables S1 and S2, available in the online Supplementary Material. Bacteria were grown overnight (approximately 18 h) in LB Lennox/ Luria broth (LB) NaCl, 5 g l^−1^, Tryptone, 10 g l^−1^ Yeast Extract, 5 g l^−1^ or on LB agar (Sigma) at 37 °C. Bacteria harbouring temperature sensitive plasmids pCP20 and pKD46 were cultured at 30 °C. Broth cultures were shaken at 150 r.p.m. for routine overnight culturing. Where required, antibiotics were added to growth media at the following concentrations: 10 µg ml^−1^, gentamicin; 50 µg ml^−1^, kanamycin; 100 µg ml^−1^, carbenicillin (all Sigma). 4-(2-amino-1,3-thiazol-4-yl)phenol (Fluorochem) was solubilised in DMSO at a stock concentration of 1M and was added to bacterial cultures to the desired final concentration.

### Mutagenesis

Mutants were constructed as per the Lambda (λ)-Red recombination protocol as detailed by Datsenko and Wanner [32]. Kanamycin (source; pKD4) and gentamicin (pMH2) resistance genes were amplified through PCR by primers which had been designed to contain homologous flanking sequences to the target genes. Amplicons were purified using the Monarch DNA and PCR Cleanup Kit (New England Biolabs) and precipitated as per the Co-Precipitant Pink (Bioline) protocol before resuspension in 4 µl molecular-grade water (Thermo Fisher). The λ-red recombination genes on the pKD46 vector were induced through the addition of l-arabinose (final concentration 10 mM, Sigma) to CFT073/pKD46 cultures for 1.5 h at 30 °C. The temperature-sensitive pKD46 plasmid was removed from CFT073 through incubation at 37 °C. Putative mutants were confirmed through PCR. All mutant strains are listed in Table S1. Plasmids vectors used to amplify antibiotic resistance cassettes for mutagenesis are listed in Table S2. All oligonucleotides used for recombination and mutant screens are listed in Table S3. To remove antibiotic resistance cassettes, mutants were transformed with pCP20, which possesses the yeast flippase gene FLP, resulting in a marker-less mutant. The protocol followed for FLP-mediated cassette removal is detailed in [32].

### RNA extraction and RT qPCR

Approximately 1×10^7^ bacterial cells were centrifuged at 12 000 ***g*** for 1 min before beginning RNA extraction as per the Monarch Total RNA Miniprep kit protocol (New England Biolabs). RNA was eluted in 30 µl nuclease-free water (Millipore) and quality and concentration were verified by Qubit and Nanodrop analysis. Nanodrop 260/280 and 260/230 scores were used to ensure RNA purity and lack of contamination by proteins or DNA. Real-time quantitative PCR (RT-qPCR) was used to quantify gene expression in *E coli*. The Luna Universal One-Step RT-qPCR Kit was used for all RT-qPCR reactions in 20 µl volumes with 10 ng RNA used as template. Standard curves were generated with fix two-fold serial dilutions of wild-type CFT073 RNA. RT-qPCR reactions were set up in triplicate in MicroAmp Fast Optical 96-well reaction plates (Applied Biosystems) and run on the StepOnePlus Real-Time PCR System (Thermo Fisher). Default ‘Fast’ RT-qPCR and melt curve settings were utilised. All oligonucleotides used for RT-qPCR are listed in Table S3. Data analysis was carried out using the StepOne software or Prism GraphPad. Housekeeping gene *rplT* was used as an internal control. Relative expression (or ‘RQ’) is calculated automatically by the instrument. RQ is the fold-change compared to the calibrator (calibrator is usually the untreated or wild-type sample – the calibrator is indicated in each figure legend). The calibrator has a RQ value of 1 and the values for the other samples are the fold-change relative to the calibrator.

### Co-visualisation of O6 and K2 by SDS-PAGE

To prepare whole-cell lysates for SDS-PAGE, 1 ml overnight or exponential cultures of CFT073 strains were centrifuged at 13 000 ***g*** and resuspended in 1X Laemelli (Sigma) to a final concentration of 10 OD_600nm_. Samples were boiled at 100 °C for 10 min and allowed to cool before adding 20 µg ml^−1^ Proteinase K (Sigma) and incubating at 56 °C for 1 h. Then 20 µl of samples were stored at −20 °C or run on a 4–20% TruPAGE Precast gel at 180V in 1X TruPAGE SDS Buffer (both Sigma). Following electrophoresis, gels were stained according to the Pro-Q Emerald 300 Lipopolysaccharide Stain Kit (Thermo Fisher) and visualised under the Quantity One (Bio-Rad) imaging system. Alternatively, gels were stained with 0.125% Alcian blue solution (Sigma) to observe total polysaccharide content.

### Western blot and densitometry

CFT073 whole cell lysates were separated by SDS-PAGE on TruPAGE precast gels as described above. Gels were transferred onto polyvinyl difluoride (PVDF) using the iBlot 2 (Thermo Fisher) for dry transfer. Membranes were blocked in 5% Bovine Serum Albumin (BSA) (Sigma) in 1X PBS supplemented with 0.1% Tween 20(0.1% PBS-T) for 1 h at room temperature (RT). Membranes were incubated in anti-O6 or anti-K2 primary antibody (Statens Serum Institut) diluted 1/500 in 5% BSA 0.1% PBS-T for 90 min at room temperature (RT). Membranes were washed three times for 10 min in 0.1% PBS-T before incubating with anti-rabbit-HRP antibody (Cell Signalling Technologies) diluted 1/10 000 in 5% BSA 0.1% PBS-T for 45 min at RT. Membranes were washed four times before staining for 5 min with Pierce ECL Western Blotting Substrate (Thermo Fisher) as per the manufacturer’s protocol. Membranes were imaged under the ImageQuant Las4000 (GE Healthcare Life Sciences). ImageStudio Lite (v 5.5.4) was utilised to relatively (no absolute values) quantify bands from Western blot .TIFF images. The quantitative values reflect the relative amount of polysaccharide as a ratio of each band relative to the gel background.

### Surface hydrophobicity

Percentage hydrophobicity was determined for CFT073 and derivatives using the protocol described in [33]. Briefly, overnight cultures were harvested by centrifugation and washed twice in 0.9% NaCl solution. Cells were standardised to an OD_600nm_ of 1 in 0.9% NaCl. A volume of 300 µl of n-hexadecane was added to 1.4 ml of the above cell suspensions before mixing by vortex. The phases were allowed to separate at room temperature for 30 min before the optical density of the aqueous phase was measured at OD_600nm_. Surface hydrophobicity was calculated as the percentage of OD extracted into the n-hexadecane phase.

### ELISA

Cultures were washed twice in PBS and standardised to OD_600_=0.1 in PBS before plating 25 µl onto a poly-L lysine treated 96-well plate (Greiner) for overnight incubation at 4 °C. The plates were centrifuged for 1 min at 10 000 ***g*** before the removal of supernatant and addition of 100 µl 0.1% v/v glutaraldehyde (Sigma) for 10 min at RT. Wells were washed three times with PBS-T before the addition of 200 µl 5% (w/v) skimmed milk (Marvel) in PBS-T. Plates were blocked for an hour at 37 °C before the addition of anti-OmpF or anti-K2 antibody diluted 1/1000 to each well for incubation at 37 °C for 1 h. Wells were washed three times vigorously with PBS-T before addition of 200 µl anti-rabbit-AP antibody (Cell Signalling Technologies) at 1/10 000 dilution to each well. Wells were washed for a final three times with PBS-T before adding 100 µl of the 1-Step PNPP (Thermo Fisher) to each well. The substrate was mixed thoroughly by gently agitating the plate at RT for 30 min. To stop the reaction, 50 µl 2M NaOH was added. Absorbance was measured at 405 nm.

### Immunofluorescence microscopy

To prepare bacterial cultures for immunofluorescence microscopy 1 ml stationary phase or exponential cultures were standardised to 0.1 OD_600nm_ and washed twice in PBS before re-suspending in 100 µl 1X PBS/ 0.01% Glutaraldehyde (Sigma). A loop-full (approx. 5 µl) of culture was spread onto a glass slide before drying at 50 °C. Then 50 µl 5% BSA solution in 0.1% PBS-Tween 20 (Sigma) was added to the slide and incubated at 37 °C for 30 min before the slide was dried as before. Once dried, 50 µl of anti-K2 or anti-O6 antibody (Statens Serum Institut) was added to the slide, diluted 1/500 in BSA solution before incubating at 37 °C for 90 min. The antibody solution was washed off the slide three times with PBS-Tween. Slides were dried again at 50 °C, prior to the addition of anti-rabbit Alexa-Fluor 594-conjugated secondary antibody (ThermoFisher) diluted 1/10 000 in 5% BSA and incubated at 37 °C for 30 min. The slide was rinsed three more times before a final drying step. A loop-full of 80% sterile glycerol (v/v) was added to the slide before the addition of the cover slip prior to visualisation and imaging under the EVOS M5000 (ThermoFisher).

### Serum killing assay

The serum sensitivity of CFT073 and mutant derivatives was examined by exposing cells to 50% normal human serum (NHS) (Biowest) and heat-inactivated serum (HIS) for 90 min at 37 °C and determining survival through viable counting as previously described [4]. Serum was heat-inactivated by heating at 56 °C for 30 min. Percentage survival was calculated as a fraction of c.f.u. ml^−1^ at T90 in NHS over c.f.u. ml^−1^ at T90 in HIS (×100). Serum resistance was measured at logarithmic (OD_600nm_ = 0.6) and stationary phase (OD_600nm_ = 3.0). Standardised assays were conducted in order to directly compare response of stationary phase and logarithmic cells to serum at equivalent cell numbers. Stationary phase cultures (OD_600_=3.0) were diluted to OD_600_=0.6 in conditioned LB (the cell-free growth supernatant of the strain).

### Minimum inhibitory concentration testing

The agar dilution method involved the addition of varying concentrations of rifampicin (Sigma) to nutrient agar medium (Sigma), prior to polymerisation. The prepared agar plates were then inoculated with 100 µl culture which was spread evenly across the plate using sterile bacteriological swabs, with standardised concentrations of overnight culture obtained through serial dilution following absorbance measurements. Minimum inhibitory concentration (MIC) was determined as the lowest concentration which inhibited bacterial growth.

### Graphing

All graphing was completed using GraphPad Prism (v.9.5.0). Graphical figures were made using Biorender (Biorender, online graphing tool).

### Statistical analysis

All statistical analysis was carried out using the GraphPad Prism software (v.9.5.0) unless otherwise stated. Statistical analysis was performed exclusively on biological replicates, wherein experiments were conducted a minimum of three (*N*=3) or four times (*N*=4). In all data, a *p* value of ≤0.05 is denoted *; a *p* value of ≤0.01 is denoted **; a *p* value of ≤0.001 is denoted ***; and a *p* value of ≤0.0001 is denoted ****.

## Results

### Mutagenesis and visualisation of the CFT073 extracellular glycome

**Fig. 1.**
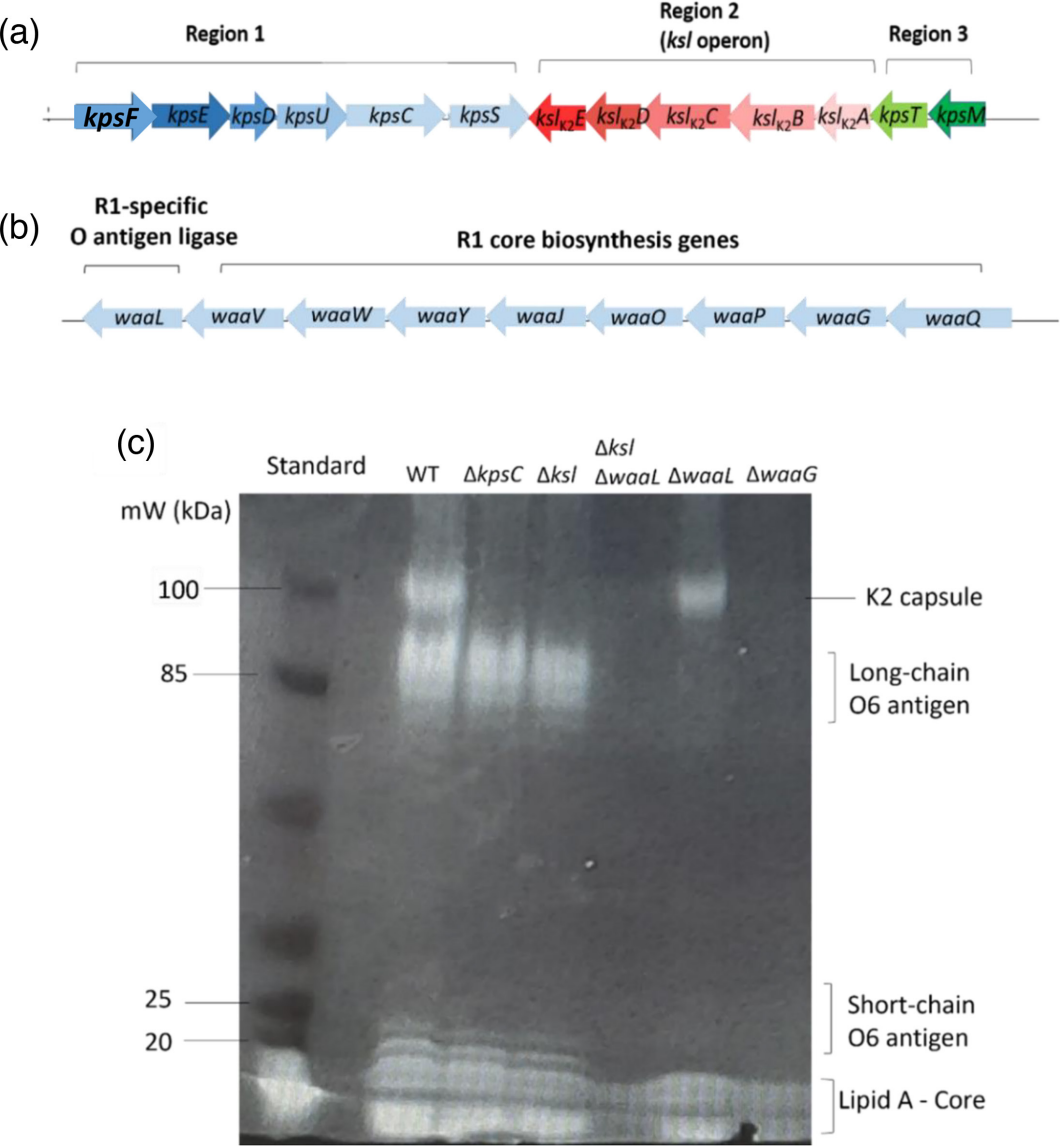
Mutagenesis and visualisation of CFT073 extracellular glycome. (**a**) and (**b**) depict the regions targeted in mutagenesis, specifically the K2 capsule gene cluster, the R1 core biosynthesis cluster, and the R1-specific O antigen ligase. (**c**) Whole cell lysates of strain CFT073 and mutant derivatives were separated by SDS-PAGE and stained with the ProQ Emerald 300 LPS stain, allowing co-visualisation of LPS and capsule.

Components of the extracellular glycome (K2 capsule, LPS outer core and O6 antigen) were mutated with specific genes essential in the biosynthesis of the polysaccharides targeted. The region two (serotype specific) *kslCDABE* operon of the K2 capsule biosynthesis cluster was targeted to make a mutant lacking the K2 capsule, Δ*ksl*, in addition to *kpsC,* a gene essential for capsule export (Fig. 1a). Outer core biosynthesis was targeted through deletion of the gene encoding the core glycosylase enzyme WaaG (Fig. 1b). Lastly, an O6 antigen-deficient mutant was made through deletion of the O antigen ligase gene, WaaL (Fig. 1b). Whole cell lysates of wild-type and the panel of glycome mutants were separated by SDS-PAGE and stained with ProQ Emerald 300 (see Fig. 1c). Fig. 1c shows the stained extracellular glycome of CFT073, consisting of the full-length, smooth LPS molecule and the high molecular weight K2 capsule, as well as the mutants lacking in the corresponding bands to their mutation, e.g. the *waaL* mutant possesses capsule and lipid A-core but is deficient in O6 antigen due to the deleted O antigen ligase. Interestingly, *waaG* mutants do not appear to possess K2 capsule as evidenced by the stained SDS-PAGE gel.

### O antigen and outer core mutants display decreased capsule

**Fig. 2.**
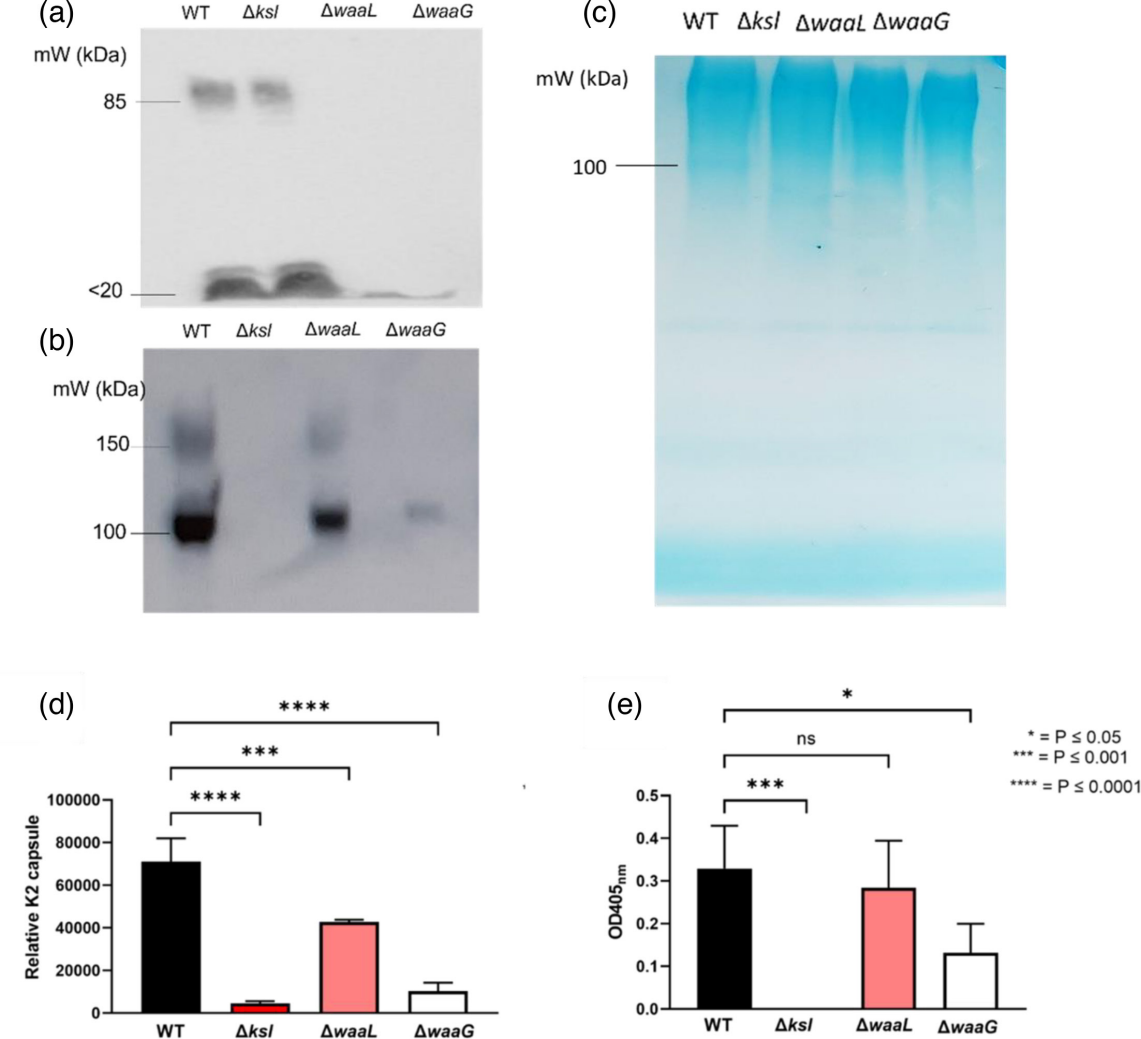
Western blot analyses depict decreased capsule in LPS mutants. Whole cell lysates of strain CFT073 and derivative glycome mutants were separated by SDS-PAGE and subject to (**a**) Western blot analysis of the O6 antigen, using an O6-specific antibody, (**b**) Western blot analysis of the K2 capsule, using a K2-specific antibody and (**c**) a 0.125% Alcian blue total polysaccharide stain, which served as a loading control. D and E represent (**a**) Densitometric analysis of Western blots and (**b**) ELISA allowing for quantification of K2 capsule. *N*=4 for both assays. WT=Wild-type CFT073. Statistical significance calculated by One-way ANOVA and Dunnett’s multiple comparisons.

Next, a protocol for efficiently blotting the high molecular weight polysaccharides was optimised. Western blot analysis depicted full LPS in strain CFT073 wild-type and *ksl* mutant whole cell lysates, but not in *waaL* or *waaG* mutant preparations, as expected (Fig. 2a). Similarly, K2 capsule was detected in Western blot analysis of wild-type, *waaL* and *waaG* whole cell preparations, but was not detected in the *kslABCDE* operon mutant, as seen in Fig. 2b. Interestingly, there appears to be less visible capsule in O antigen and *waaG* mutants. The same cell preparations used in Fig. 2a and b were stained with 0.125% Alcian blue (Fig. 2c) following separation by SDS-PAGE to serve as a loading control for the standardised whole cell lysates, showing equivalent polysaccharide in each lane. Next, the apparent reduction in K2 capsule levels observed in LPS mutants was quantified by densitometry (Fig. 2d) and ELISA (Fig. 2e). Densitometry was performed on *N*=4 biological replicates of the Western blot performed on whole cell lysates depicted in Fig. 2b. The densitometric quantification of K2 capsule showed that *waaL* mutants possess approximately 40% less K2 capsule than wild-type, as detected by Western blot, a statistically significant reduction. Similarly, *waaG* mutants displayed 86% less K2 capsule than wild-type. Interestingly, ELISA quantification of K2 capsule showed significantly decreased capsule in *waaG* mutants compared to wild-type (*P*≤0.05), whilst *waaL* mutants displayed no significant reduction in capsule levels compared to wild-type (*P*=0.8). The reduction in capsule seen for *waaG* and *waaL* mutants was less pronounced when examined by ELISA in comparison to Western blot.

### LPS mutants have increased capsule in culture supernatants

**Fig. 3.**
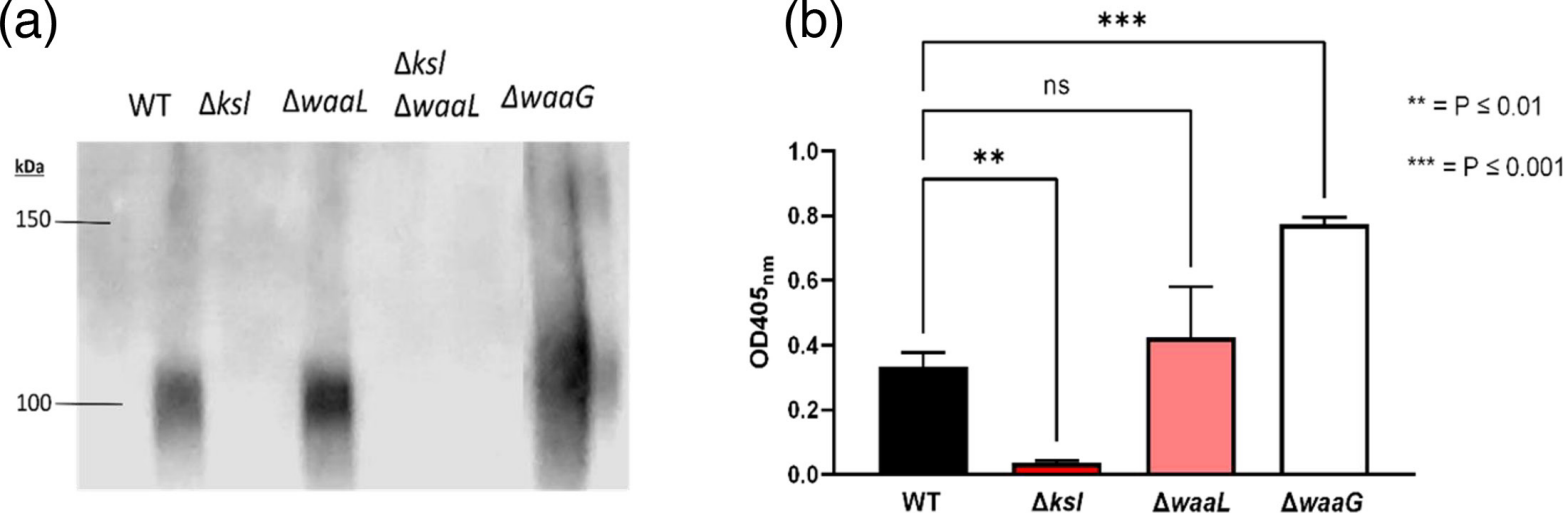
LPS mutants possess increased K2 in the supernatant. (**a**) Western blot analysis of K2 capsule in stationary phase supernatant taken from bacterial cultures and concentrated ten-fold. (**b**) ELISA quantification of K2 capsule in stationary phase supernatant of CFT073 and mutants. *N*=4 for both assays. WT=Wild-type CFT073. Statistical significance calculated by One-way ANOVA and Dunnett’s multiple comparisons.

Due to the unexpected findings depicted in Fig. 2e, whereby ELISA quantification showed that LPS mutants did not display pronounced reductions in capsule, it was hypothesised that the anti-K2 antibodies were binding secreted K2 capsule. The ELISA protocol used in this study includes the incubation of cells prior to fixing, allowing for the secretion of molecules, whilst whole-cell lysate preparation for Western blot involves the removal of culture supernatant prior to SDS-PAGE. Culture supernatants taken from strain CFT073 and mutant derivatives were concentrated ten-fold prior to SDS-PAGE and Western blot analysis. As depicted in Fig. 3a, the anti-K2 Western blot confirmed K2 capsule in the supernatant of wild-type, *waaL* and *waaG* mutants. Next, the secreted K2 in culture supernatants was quantified by ELISA, as shown in Fig. 3b. The *waaL* mutant displayed more secreted capsule in culture supernatant than wild-type, although this increase was not statistically significant (*P*=0.4), whilst the *waaG* mutant displayed significantly more secreted K2 than wild-type (*P*=0.0001). These results indicate that the reductions in capsule seen in LPS mutants occurs due to changes in surface composition by virtue of truncated LPS, which has an adverse effect on capsule surface retention.

### Decreased capsule seen in LPS mutants is post-transcriptional

**Fig. 4.**
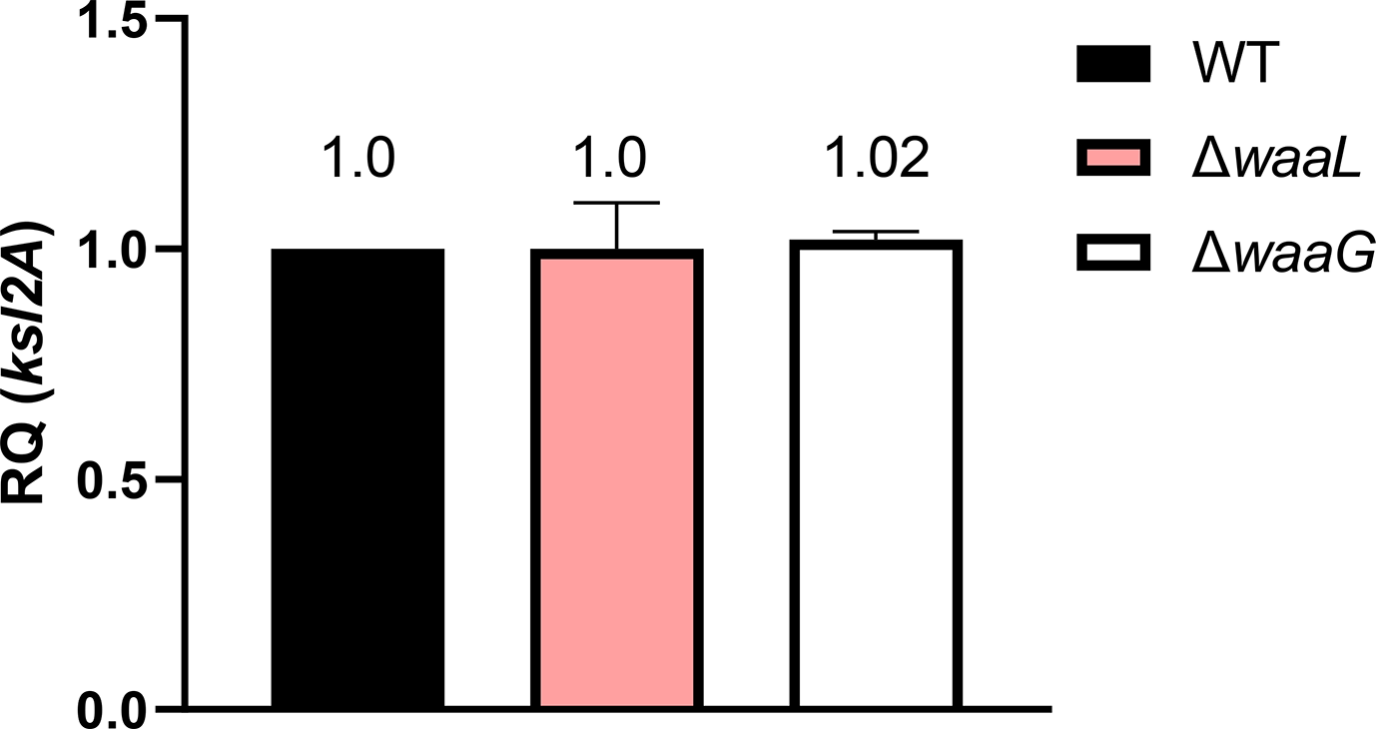
Decreased capsule in LPS mutants occurs post-transcriptionally. RNA was extracted from stationary phase cultures grown under standard lab conditions (LB, 37 °C) and *ksl2A* transcript levels were compared. Data analysis and relative expression calculation was carried out automatically on the StepOne software, with housekeeping gene *rplT* used as an internal control. Statistical analysis by One-way ANOVA and Dunnett’s multiple comparisons. WT=Wild-type CFT073. *N*=4 biological replicates.

Following the observation that LPS mutants display reductions in surface capsule and increased secreted capsule, it was hypothesised that the dependence of capsule on full-length LPS was post-transcriptional and related to changes in surface hydrophobicity. RT-qPCR was conducted on RNA extracted from strain CFT073 wild-type, as well as *ksl* (not shown), *waaL* and *waaG* mutants to compare relative expression of the *kpsT/ksl2A* genes. As shown in Fig. 4, *waaL* and *waaG* mutants displayed no changes to region 2 *kpsT/ksl2A* gene transcription when compared to wild-type. A similar analysis exploring region 1 (*kpsC*) expression showed no change to *kpsC* expression in LPS mutants when compared to wild-type (S5). Additionally, region 3 genes are transcribed with region 2 as an operon, and so region 3 was not explored [30]. Overall, these results indicate that there are no downstream effects on K2 gene expression in LPS mutants.

### Complementation of LPS restores capsule levels

**Fig. 5.**
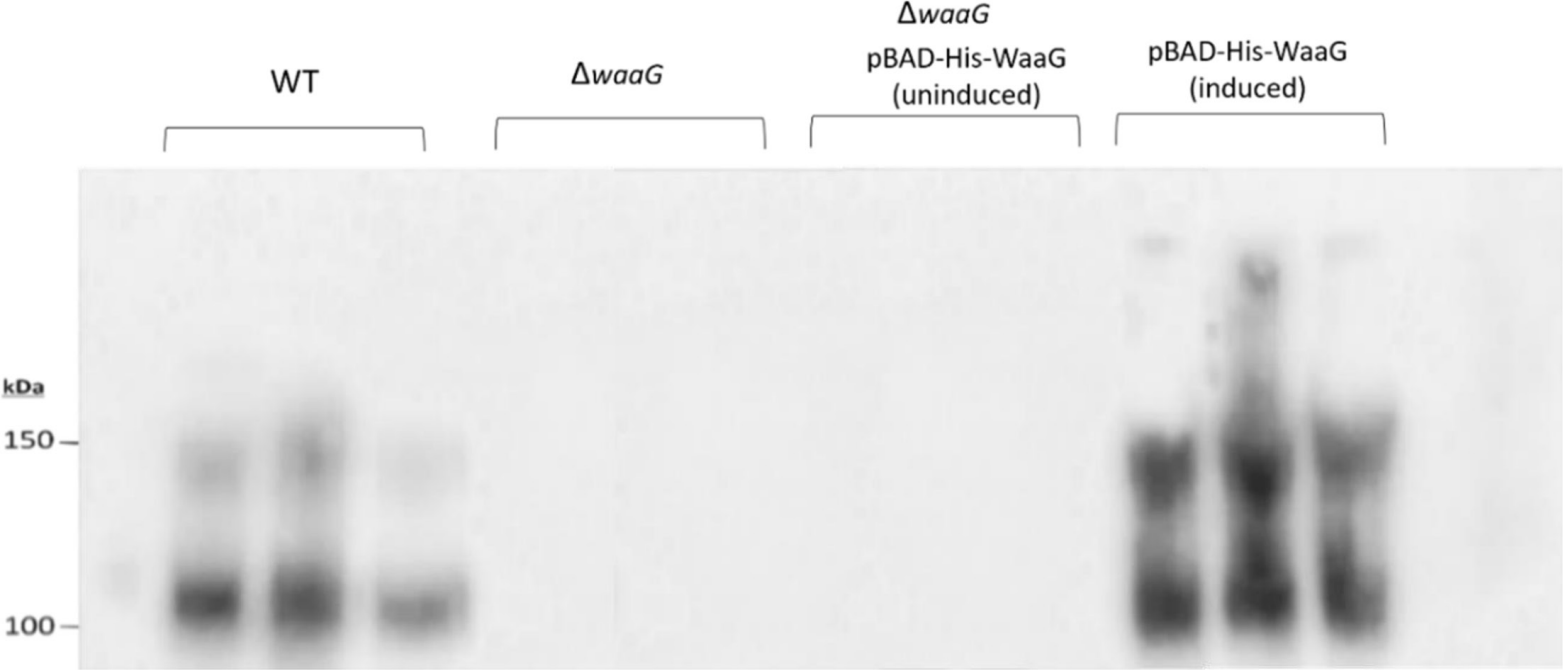
Complementation of Δ*waaG* restores capsule. Western blot analysis of K2 capsule in wild-type, *waaG* mutant and complemented mutant under inducing conditions (0.2% w/v l-arabinose). *N*=3 biological replicates performed and depicted in the above figure. WT=Wild-type CFT073.

In order to confirm that the synergy between LPS and capsule is related to surface interactions and is not due to downstream or compensatory mutations, the *waaG* mutant was complemented with pBAD-His-WaaG, a vector with inducible *waaG* expression. Fig. 5 shows Western blot analysis of K2 expression in wild-type and Δ*waaG,* in addition to the complemented *waaG* mutant under induced (+ l-arabinose) and un-induced conditions. As seen below, the *waaG* mutant complemented with pBAD-His-WaaG post-induction with l-arabinose displayed increased K2 capsule, confirming that the *waaG* mutation is non-polar and that the synergy between LPS and capsule occurs due to changes at the cell surface. The O6 antigen mutant deficient in *waaL* also displayed restored K2 levels comparable to that of wild-type upon complementation of the *waaL* gene (data not shown), indicating that both moieties of LPS (O antigen and core) interact with K2 to facilitate surface retention.

### LPS mutants display changes in surface hydrophobcity

**Fig. 6.**
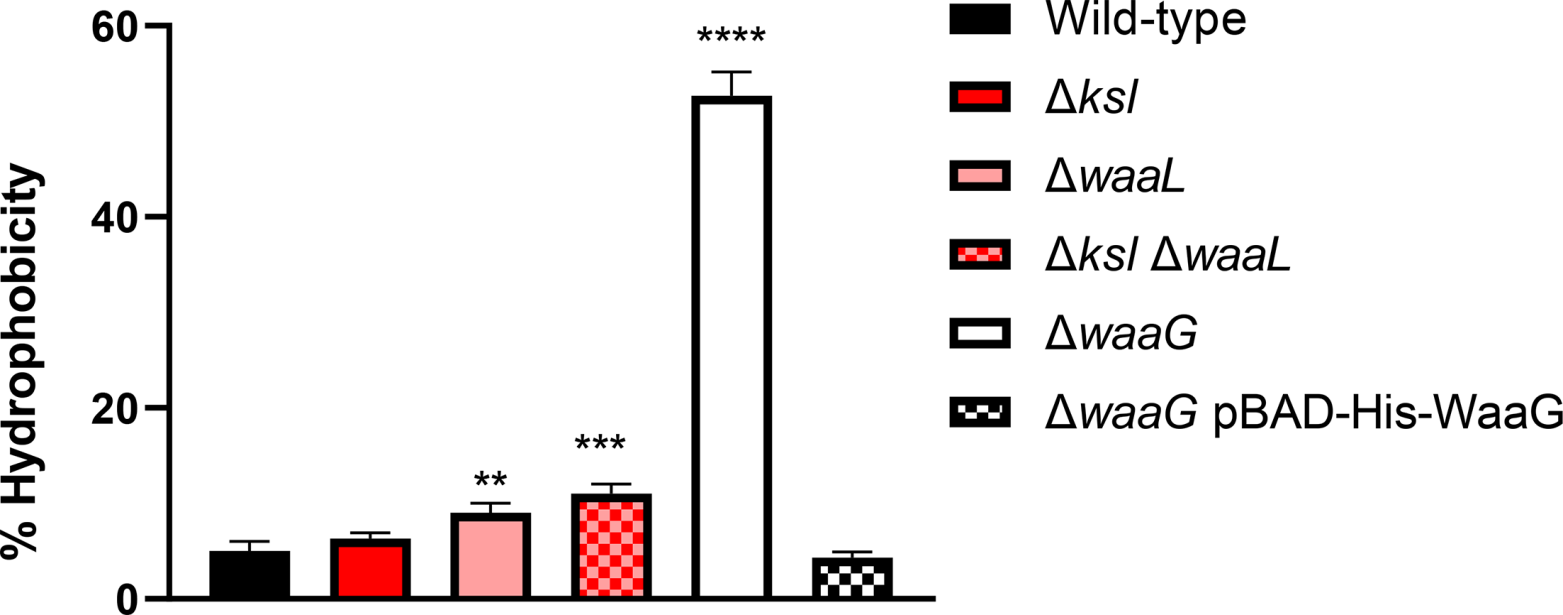
Increased surface hydrophobicity in LPS mutants. Surface hydrophobicity was determined via the n-hexadecane binding assay, as detailed in [33]. Percentage hydrophobicity was calculated by dividing the OD_600nm_ of the aqueous phase by the starting OD_600nm_ of 1 (×100), following the incubation of cells with n-hexadecane. *N*=3. Statistical significance calculated by One-way ANOVA followed by Dunnett’s multiple comparisons. Wild-type=Wild-type CFT073. ** = *P*≤0.01, *** = *P*≤0.001, **** = *P*≤0.0001.

Surface hydrophibicity of the ExPEC derivatives was determined using the n-hexadecane hydrophobicity assay, as detailed in [33], with results depicted in Fig. 6. LPS mutants including Δ*waaL*, Δ*waaL* Δ*ksl* and Δ*waaG* displayed significant changes in surface hydrophobicity relative to wild-type CFT073. The outer core mutant deficient in *waaG* displayed the most significant change in surface hydrophobicity, in fact, the strain displayed an approximate ten-fold increase in hydrophobicity (52% cells in aqueous phase) relative to wild-type (5% cells). These results demostrate the significant changes which occur to CFT073 surface hydrophobicity in the absence of LPS, correlating with reduced capsule retention.

### Outer core mutants possess a punctate K2 localisation pattern

**Fig. 7.**
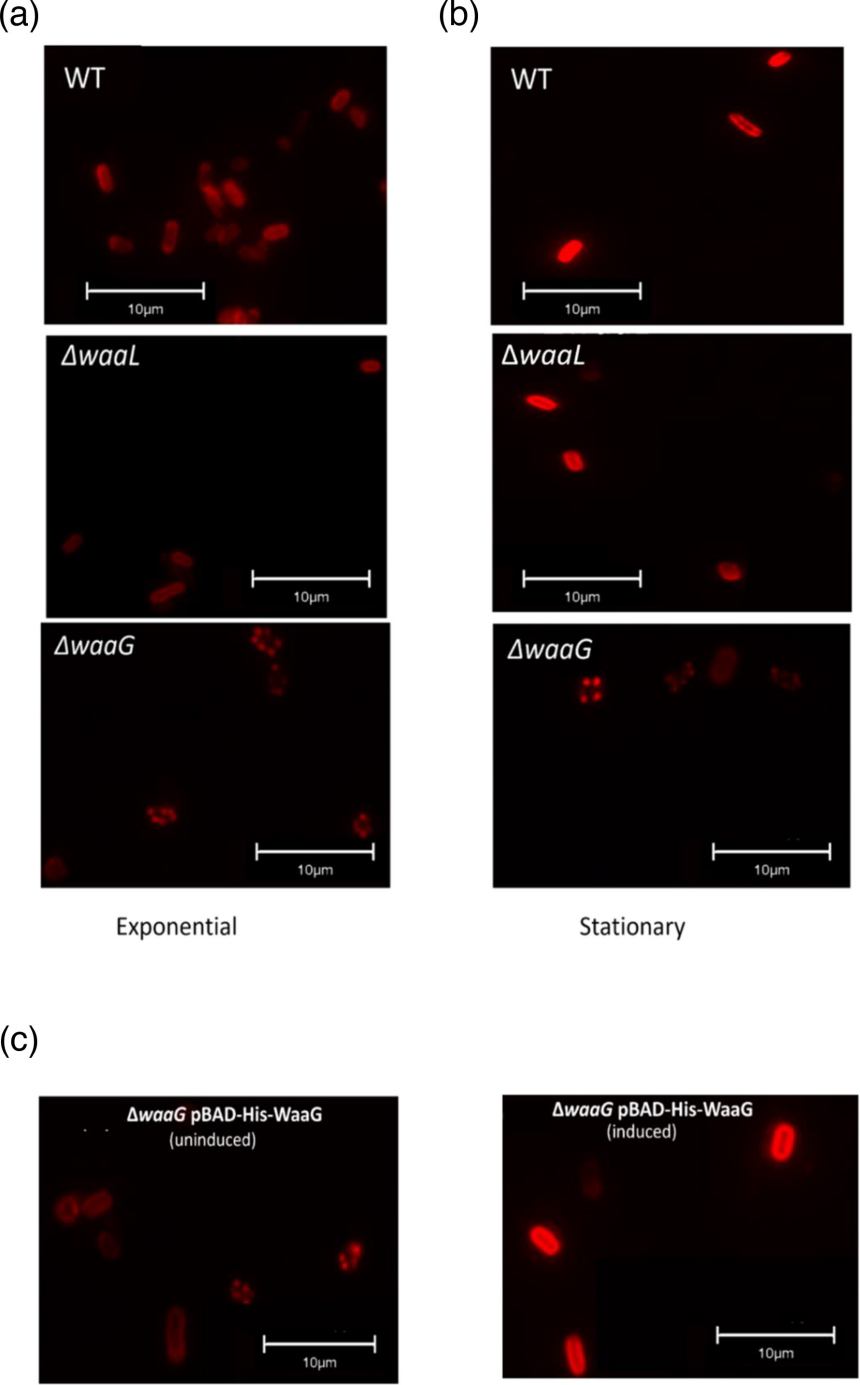
*waaG* mutants display punctate capsule pattern. (**a**) Exponential, (**b**) stationary phase and (**c**) stationary phase induced / uninduced CFT073 cultures were probed with anti-K2 antibody and anti-rabbit conjugated to AlexaFluor594 to detect surface capsule. *N*=4 biological replicates. *N*=1 shown per growth phase. WT=CFT073 Wild-type. Uninduced=grown in LB. Induced=grown in LB supplemented with 0.2% w/v l-arabinose.

Despite the presence of decreased capsule in O6 antigen and outer core mutant derivatives of CFT073, as seen in Fig. 2 (Western blot on whole-cell lysates) the mutants do still possess some K2 capsule. In order to discern whether this capsule is intracellular or on the cell surface, immunofluorescence microscopy was performed on exponential and stationary phase CFT073 cultures as depicted in Fig. 7. These results showed that at both exponential and stationary phase, the *waaG* mutants display a punctate capsule pattern, compared to the homogenous coating of capsule seen on the surface of wild-type cells. Interestingly, despite *waaL* mutants displaying decreased capsule, the immunofluorescence microscopy showed that surface localisation of the K2 capsule is comparable to that of the wild-type capsule, although with reduced signal. These results indicate changes to the localisation of capsule in *waaG* mutants in addition to the decreased retention of surface capsule, whilst *waaL* mutants solely display decreased surface capsule without changes to localisation patterns. Complementation of the *waaG* mutants with pBAD-His-WaaG resulted in restored capsule comparable to wild-type as well as full length LPS, as shown in Fig. 5, and so it was hypothesised that complemented mutants would also display restored capsule localisation and coverage of the bacterial cell. Immunofluorescence microscopy was also carried out on wild-type (not shown) and Δ*waaG* cells complemented with pBAD-His-WaaG under inducing conditions (0.2% l-arabinose – WaaG expression is under regulation of the P*araBAD* promoter), as well as non-inducing conditions as a control. Fig. 7c depicts *waaG* mutants complemented with the pBAD-His-WaaG vector under non-inducing conditions (i.e. no l-arabinose added). The cells display the same punctate capsule pattern observed previously. Interestingly, *waaG* mutants complemented with the pBAD-His-WaaG vector under inducing conditions display capsule localisation on the cell surface comparable to that of the wild-type.

### Contribution of glycome to serum resistance

It has been shown that the K2 capsule and O6 antigen of ExPEC strain CFT073 confer resistance to serum [5,6, 34]. However, a strain CFT073 mutant deficient in both K2 and O6 had not been explored in order to estimate the full contribution of the extracellular glycome to serum resistance. Table 1 depicts the percentage survival of CFT073 and derivatives following exposure to 50% NHS. Wild-type was largely resistant to 50% NHS at exponential growth phase (85% survival compared to wild-type cells exposed to HIS). Capsule mutant Δ*ksl* displayed a significant reduction in serum resistance (4% survival) in comparison to wild-type (*P*<0.0001) during exponential growth. Similarly, O antigen deficient Δ*waaL* in exponential phase displayed significantly reduced survival in 50% NHS (0.01% survival) in comparison to the wild-type (*P*<0.0001). Strikingly, the double mutant Δ*ksl* Δ*waaL* was almost entirely sensitive to 50% NHS during exponential growth phase, with only 0.000038% of cells surviving. This reduction in survival was statistically significant when compared with the percentage survival of wild-type CFT073 (*P*<0.0001). Only 0.009% of exponential *waaG* survived when exposed to 50% NHS, a significant reduction from wild-type survival (*P*<0.0001). Furthermore, the survival of stationary phase CFT073 strains after exposure to 50% NHS for 90 min was examined. Stationary phase wild-type cultures displayed resistance to 50% NHS with 90% of cells surviving. The slight reduction in survival of exponential (85%) in comparison to stationary phase (90%) wild-type CFT073 was not significant (*P*=0.4). Stationary phase Δ*ksl* and Δ*waaL* mutants were significantly more sensitive to serum than wild-type, with 4 and 0.2% survival, respectively (*P*<0.0001 for both mutants relative to wild-type). Moreover, the double mutant, Δ*ksl* Δ*waaL*, was again, exquisitely sensitive to 50% NHS during stationary phase (0.0005% survival, *P*<0.0001, relative to wild-type). Only 0.1% of *waaG* mutants survived in 50% NHS at stationary phase growth, which was a significant reduction compared to wild-type (*P*<0.0001). Interestingly, there was a significant increase in the survival of *waaL* mutants during stationary phase compared with exponential growth, *P*=0.0346. Overall, these data indicate that LPS and capsule are the major determinants of serum resistance, as a double mutant deficient in O antigen and capsule synthesis is almost entirely sensitive to serum, with no significant difference in survival during exponential and stationary phase serum resistance between this mutant and a K-12 serum sensitive control, DH5α (data not shown as DH5α cultures display 0% survival after exposure to 50% NHS for 90 min).

**Table 1.** Extracellular glycome mutants are sensitive to 50% NHS. Percentage survival was calculated as a fraction of c.f.u. ml^−1^ at T90 in NHS over c.f.u. ml^−1^ at T90 in HIS (×100). *N*=4 for both assays. WT=Wild-type CFT073. Statistical significance calculated by Two-way ANOVA and Tukey’s multiple comparisons

	Survival in 50% NHS	
**Strain**	**Exponential**	**Stationary**
Wild-type	85	90
Δ*ksl*	4*	4*
Δ*waaL*	0.008**	0.19**
Δ*ksl* Δ*waaL*	0.00004**	0.0005**
Δ*waaG*	0.0006**	0.1125**

### WaaG inhibition results in decreased surface K2 capsule

**Fig. 8.**
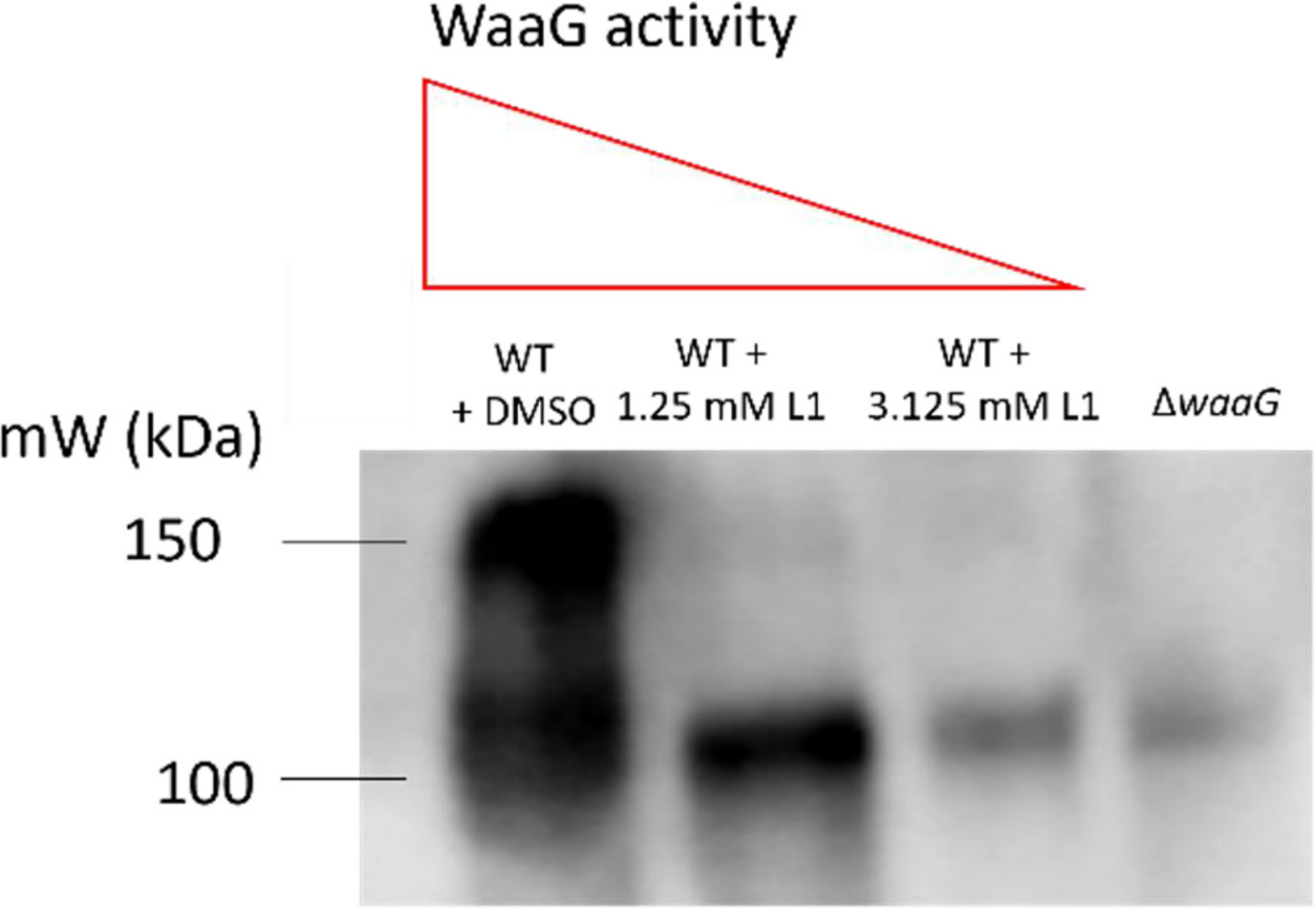
WaaG inhibition reduces K2 capsule. Whole cell lysates of the WaaG deficient mutant Δ*waaG* and wild-type CFT073 supplemented with DMSO or L1 at concentrations of 1.25 or 3.125 mM were separated by SDS-PAGE and probed with anti-K2 for Western blot analysis of LPS and capsule. *N*=3 conducted, *N*=1 shown.

Published works by Muheim *et al*. identified a WaaG inhibitor with *in vitro* activity towards purified LPS, 4-(2-amino-1,3-thiazol-4-yl)phenol (known as L1) with IC_50_=1.0 mM [35]. Due to the role for WaaG in capsule retention identified in this study (by virtue of its contribution to biosynthesis of full-length LPS and subsequently, surface hydrophobicity), it was hypothesised that L1 could be used to reduce surface association of K2 capsule with the cell. It was expected that with increased concentrations of L1, there would be dose-dependent reductions in WaaG activity, which may result in reduced or altered LPS, and thus, less capsule retained. Concentrations of L1 at 1.25 mM and 3.125 mM were explored for the inhibition of WaaG in live bacterial cultures of CFT073. L1 was solubilised in DMSO and so cultures with added DMSO, along with the *waaG* mutant were used as a control. As seen in Fig. 8, the addition of L1 to cultures results in a concentration-dependent decrease in capsule (A), in addition to changes in LPS quantity and electrophoretic mobility (Fig. S4). Taken together, the above results show that L1 can reduce capsule levels through LPS inhibition, and also displays its inhibitory activity on live bacterial cells and not just purified LPS, as had been explored in [35].

### LPS/capsule synergy can be exploited to reduce serum resistance

**Fig. 9.**
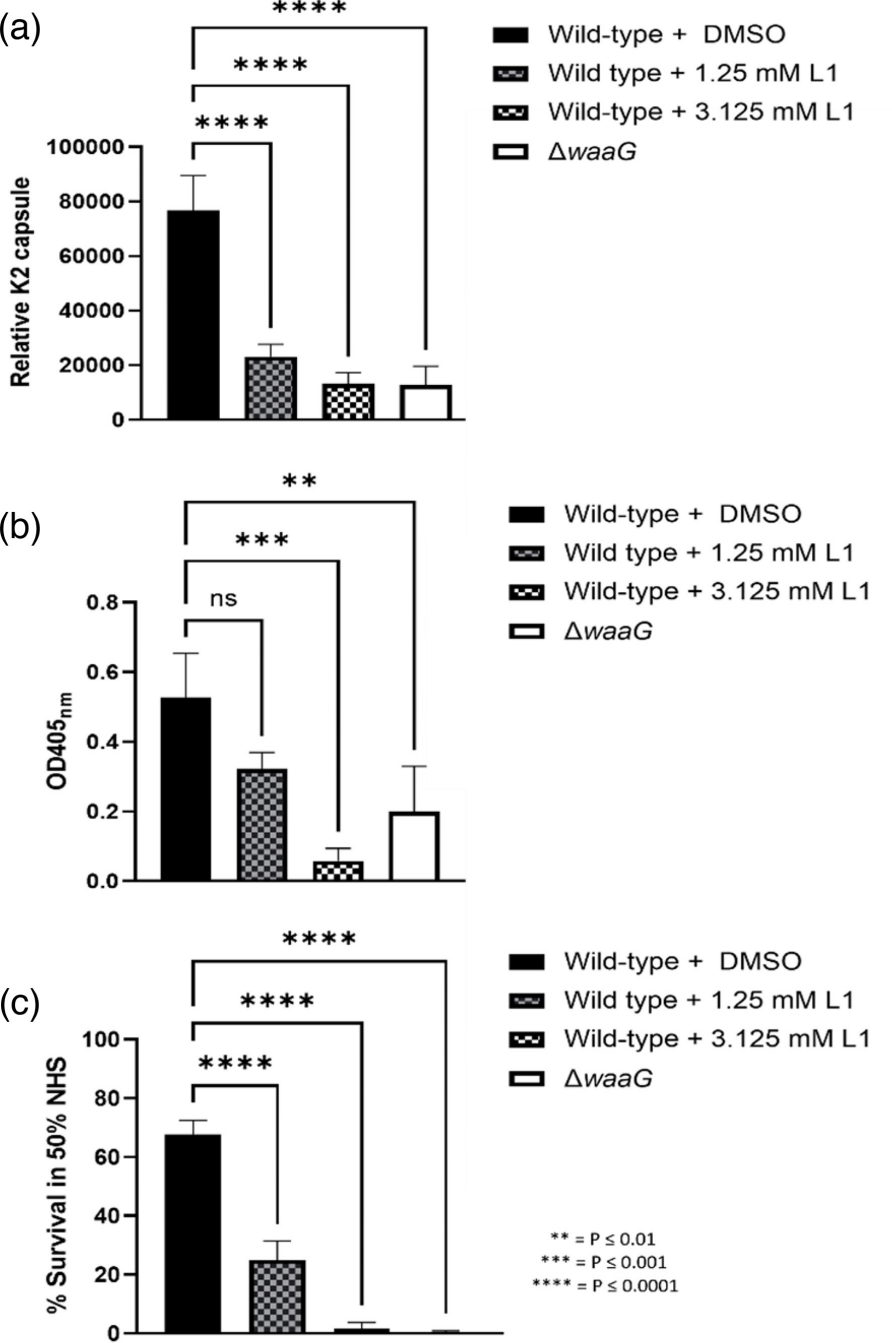
Implications of WaaG inhibition on capsule levels and serum resistance. (**a**) Densitometry performed on anti-K2 Western blots, *N*=3. (**b**) ELISA examining K2 levels conducted on wild-type CFT073 +/-the addition of L1, in addition to the *waaG* mutant, *N*=3. (**c**) Serum killing assay, percent survival calculated as a fraction of c.f.u. ml^−1^ at T90 in NHS over c.f.u. ml^−1^ at T90 in HIS (×100). *N*=4. Statistical significance calculated by One-way ANOVA and Dunnett’s multiple comparisons.

The decreases in K2 capsule levels in cultures exposed to the WaaG inhibitor L1 were quantified by (A) densitometry performed on Western blots and (B) ELISA and compared to the wild-type (+DMSO) and the *waaG* mutant. Fig. 9a depicts densitometric quantification of K2 capsule in *N*=4 Western blots, demonstrating that wild-type +L1 at 1.25 mM and 3.125 mM display significant reductions to capsule levels, compared to wild-type +DMSO (*P*<0.0001 for both). Interestingly, the addition of 3.125 mM L1 decreased capsule to just 17% of wild-type levels, which was comparable to the amount of K2 displayed by the *waaG* mutant (*P*>0.9999). ELISA quantification of capsule (Fig. 9b) mirrored those seen in the densitometric analysis, where the addition of L1 decreased K2 levels when compared to wild-type +DMSO in a concentration-dependent manner (1.25 mM L1; *P*=0.07, 3.125 mM L1; *P*=0.0009). Finally, it was determined whether the changes to the glycome mediated by L1 would alter the resistance of CFT073–50% human serum (NHS). As shown in Fig. 9c, the addition of L1 resulted in significant reductions to CFT073 serum survival in a concentration-dependent manner (*P*≤0.00001 for wild-type +L1 at 1.25 and 3.125 mM, as well was the *waaG* mutant) when compared to the wild-type +DMSO.

## Discussion

### Visualisation of CFT073 glycome

To survive in serum, bacteria must evade the host immune system including the components of the complement system. Resistance to killing by serum is achieved (in part) by the extracellular glycome of polysaccharides such as LPS, colanic acid and capsule, which act as steric barriers to the complement system, the preliminary obstacle faced by ExPEC upon entry into the bloodstream [5,6]. To further characterise the role of the extracellular glycome in CFT073 serum resistance, a panel of mutants deficient in extracellular polysaccharides O6 antigen, capsule and the outer core oligosaccharide were made. The extracellular glycome profiles of CFT073 wild-type and mutant derivatives were visualised and analysed using techniques such as SDS-PAGE, Western blot, and ELISA. As depicted in Fig. 1c, LPS staining of whole cell lysates separated by SDS-PAGE allows for the visualisation of the extracellular glycome components O6 antigen and K2 capsule simultaneously, which marks the first co-visualisation of these serotype-specific polysaccharides on an SDS-PAGE gel. Moreover, the stained SDS-PAGE gels depicted the larger ‘molecular weight’ of K2 in comparison to O6 antigen, with approximately *N*=40 units to *N*=25 for the O6 antigen. The results of the SDS-PAGE gels were reproducible, including when conducted at different growth stages, indicating the length and size of these polysaccharides are not phase variable. Western blot analyses of the K2 capsule (such as that show in Fig. 2b) depicted two bands which resolve at approximately 100 and 150 kDa, an observation previously made in [26], which was also reproducible regardless of cell concentration or growth phase.

### Role of extracellular glycome in serum resistance

The relative survival in 50% NHS of CFT073 and glycome mutants at exponential and stationary phase was examined in this study. During exponential and stationary phase growth, both CFT073 Δ*ksl* and CFT073 Δ*waaL* displayed reduced survival rates in serum in comparison to wild-type CFT073. Interestingly, the *waaL* and *waaG* mutants were more sensitive to serum than the *ksl* mutant at both growth phases, although this is likely due to the fact that CFT073 LPS mutants also display significant reductions in surface K2 capsule. Interestingly, the double O antigen/capsule mutant, Δ*ksl* Δ*waaL* displayed the highest drop in survival relative to wild-type, with the c.f.u. ml^−1^ counts of 2/4 exponential biological replicates being reduced to 0, indicating complete killing of the strain by complement. These data reinforce that the O antigen and capsule are the two main determinants of serum resistance in ExPEC strain CFT073, and that exploiting their synergy is a potential therapeutic strategy.

### Synergy between the R1 core, O6 antigen and the K2 capsule

The anti-K2 Western blots conducted on whole cell lysates depicted an apparent reduction in K2 capsule in *waaL* and *waaG* mutants, compared to wild-type CFT073. This reduction was quantified through densitometry and ELISA (Fig. 2d and e). Interestingly, CFT073 *waaL* mutants displayed on average a 50% reduction to capsule levels, whilst *waaG* mutants displayed only 10–12% of wild-type capsule levels, as determined by densitometry. These results imply that both O antigen and the LPS core are interacting with K2 to contribute to its retention at the cell surface. Mutants deficient in *rfaH* and *wzy* also displayed reductions to K2 capsule, further reinforcing that the interactions between LPS and capsule retain K2 at the surface and the genes are not involved in cross-regulation or a feedback loop (data not shown).

The reliance of capsule on LPS core moieties has previously been observed in *E. coli* but only in a K1:O18 background in strain RS218, and in a K5 serotype strain [25,27]. Thus, the findings of this study confirm that the interactions between LPS and capsule are not just K1 and K5 specific, but also occur in K2 *E. coli* and likely other K types. Similarly, in *Klebsiella pneumoniae*, an intact LPS core molecule has been shown to contribute to capsule retention for several different O and K serotypes in strains 52145 (O1:K2), DL1 (O1:K1) and C3 (O8:K66) [29]. However, in both of the aforementioned studies (*E. coli* K1 and *Klebsiella*), *waaL* mutants did not display decreased capsule relative to wild-type *E. coli* and *Klebsiella pneumoniae*, implicating solely the charge of the core molecule in capsule retention for these prototypic strains [27,29]. Perhaps the interaction of O6 with K2 in CFT073 is strain-specific or serotype-specific, whilst the core-capsule interaction is more conserved across different strains and serotypes. Supporting this hypothesis is a study conducted on a different *Klebsiella pneumoniae* strain to those studied in [29], in which *Klebsiella pneumoniae* B5055 (O1:K2) *waaL* mutants displayed a 50% reduction to surface capsule levels [28]. As different core types and capsule types will be composed of different sugar structures with different modifications (such as phosphorylation), it is not unexpected that whilst the synergy between LPS and capsule is conserved, the specific interactions involved in the surface retention (e.g. in CFT073 K2/core as well as K2/O6 interact vs in RS218 solely K1/core) differ from strain to strain depending on the serotype.

The R1 core molecule (shared between CFT073 and RS218 – as well as most other ExPEC isolates) is highly phosphorylated, giving rise to the net-negative charge of the core [10]. Due to this negative charge, the association of divalent cations such as Mg^2+^ and Ca^2+^ cross-link LPS, provide membrane stability, as well as serve as a Ca^2+^ reservoir for RTX family toxins like HlyA [36]. RS218 (K1:O18) mutants deficient in the enzymes catalysing the phosphorylation of the R1 core (such as *waaY* – unphosphorylated HepII) display no structural changes to LPS other than changes to its net charge due to the lack of phosphate groups, yet display only 25% surface capsule compared to wild-type RS218 [27]. Such findings validate the hypothesis that the interactions between LPS and capsule are ionic interactions which result in capsule retention. Additionally, this study has shown that LPS mutant derivatives, particularly *waaG* mutants, display significant changes to surface hydrophobicity which further implicates the importance of surface interactions in capsule retention.

Based on the findings from the *E. coli* K1 paper [27], and the findings depicted in Fig. 4 which demonstrated that the changes to capsule in LPS mutants were post-transcriptional, it was hypothesised that the capsule not retained on the cell surface was present in the supernatant. Analysis of culture supernatants depicted that (a) under normal conditions there is some K2 capsule secreted into the supernatant by wild-type CFT073 and that (b) *waaL* and *waaG* mutants display significantly more K2 capsule in the supernatant compared to wild-type due to the inability of these mutants to retain capsule in the absence of interactions with the outer core and O antigen (Fig. 3a and b). The presence of K2 capsule in the supernatant of wild-type CFT073 cultures can be explained by the various potential roles for free K2 capsule. The CFT073 K2 capsule has been shown to hinder biofilm formation of pathogenic competitor species such as *Pseudomonas aeruginosa, Klebsiella pneumoniae, Staphylococcus aureus, Staphylococcus epidermidis* and *Enterococcus faecalis* [19].

### Localisation of K2 capsule on the cell surface

Following the observation that *waaL* and *waaG* mutants retain decreased amounts of K2 capsule at the cell surface, extracellular K2 was visualised by immunofluorescence microscopy, as shown in Fig. 7. Interestingly, the K2 pattern on wild-type cells at exponential and stationary phase appeared, for some cells, to show more K2 capsule at the cell poles, in comparison to the equator. Other cells showed a more even distribution of K2 capsule. For all wild-type cells, however, surface capsule covered the entire cell. The observation that the cell poles possess more K2 is not unique to CFT073 and the K2 capsule, a study conducted on ExPEC strain EV136 (K1 capsule) showed that capsule export occurs evenly and randomly across the entire surface of the cell, however, the K1 located at the poles was thicker than the equatorial K1 capsule [37]. Additionally, the study found that the EV136 K1 capsule displays two qualitatively different domains of bacterial capsule height/length at the poles and at the equator, with the poles possessing longer capsule and the equators possessing shorter polysaccharide chains [37]. Similarly, another study showed that purified Group 2, K5 capsule possesses two components, denoted ‘high’ and ‘low’ molecular weight components [38]. These results provide reason for the difference in signal at the poles in some of the wild-type immunofluorescence microscopy images shown in Fig. 7, yet also provide an explanation for the two bands at approximately 100 kDa and 150 kDa visualised upon Western blot analysis of the capsule (such as in Fig. 2b).

### Exploitation of LPS/capsule synergy reduces surface capsule and resistance to serum

Muheim *et al*. showed that an aminothiazole molecule 4-(2-amino-1,3-thiazol-4-yl)phenol inhibits WaaG glycosyltransferase activity *in vitro,* preventing the outer core assembly and thus, O antigen attachment of purified LPS. The compound itself may not be appropriate to administer to humans due to potential toxicity (PubChem ID 346926) but could serve as a derivative for similar compounds. Alternatively, other natural or synthetic compounds which bind and inhibit WaaG could be explored. Regardless, this study showed that the inhibitor was effective in preventing some of the WaaG activity in CFT073, thereby affecting LPS quantity, integrity and subsequently K2 capsule association. Additionally, L1 treatment of CFT073 resulted in increased sensitivity to 50% human serum, whilst viability was unaffected (Fig. S6). Thus, a derivative compound or alternative WaaG inhibitor with higher affinity for *E. coli* WaaG could be considered as a therapeutic strategy, as inhibition of the glycosyltransferase, as we have shown, has potential to reduce ExPEC virulence through preventing O antigen and K2 capsule association with the cell [27]. However, the findings of this study require exploration *in vivo* to ascertain whether the exploitation of LPS/capsule synergy as a therapeutic strategy is as effective in an infection model. Moreover, alternative approaches such as phage therapy could be considered, as many phages target the extracellular glycome, and several K/O antigens are correlated specifically with ExPEC infections [39,40]. For example, O antigen depolymerases could be isolated and cloned from phage for therapeutic use, which would also result in decreased capsule and overall virulence [41]. Targeting the glycome through phage therapy or inhibition of LPS biosynthesis will do little to affect the microbiome and so again, are two promising avenues of research.

## Conclusions

**Fig. 10.**
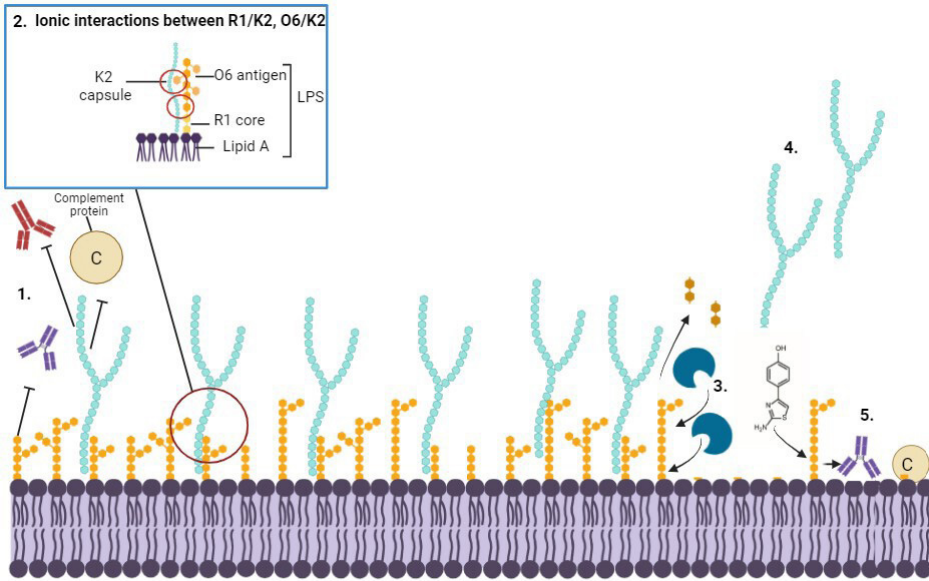
Summary of interactions between LPS and K2 and implications. Graphical depiction of the CFT073 outer leaflet, including extracellular polysaccharides LPS and K2 [42]. Under normal conditions, LPS O antigen and the K capsule provide steric barriers to antibodies and the complement system, enabling disseminated infection and sepsis [43]. The K2 capsule is retained at the cell surface through interactions with the O6 antigen and the R1 outer core, as well as Braun’s Lpp (not shown) [44]. LPS-capsule synergy can be exploited through treatment with LPS depolymerases (blue) or WaaG inhibitors such as L1 [7]. Depolymerisation of LPS or WaaG inhibition result in increased cell permeability and decreased capsule association, allowing [45] access to the bacterial cell surface for antibody binding and complement protein deposition.

The synergy between LPS and capsule observed in *E. coli* K1, K5 and several serotypes of *Klebsiella pneumoniae* also exists in strain CFT073 which possesses the R1 core, O6 antigen and K2 capsule. Due to the existence of the interactions between LPS and capsule across other species and strains, the synergy may be conserved across other members of the *Enterobacteriales* and requires exploration. This synergy has potential to be therapeutically targeted in order to sensitise bacteria to complement and thus, reduce overall virulence. The CFT073-specific interactions between components of the extracellular glycome is graphically summarised in Fig. 10.

## supplementary material

10.1099/mic.0.001493 Uncited Supplementary Material 1.

## Abbreviations

AMR: antimicrobial resistance
CFU: colony forming units
E. coli: Escherichia coli
ExPEC: extra-intestinal pathogenic Escherichia coli
HIS: heat-inactivated serum
LPS: lipopolysaccharide
NHS: normal human serum
ST: sequence type
WT: wild-type
