# Supplementary material for: Synergy between Group 2 capsules and lipopolysaccharide underpins serum resistance in extra-intestinal pathogenic Escherichia coli
Source: Microbiology (Reading). 2024 Aug 23;170(8):001493. doi: 10.1099/mic.0.001493 (PMC11342863; doi:10.1099/mic.0.001493)
Supplement: Uncited Supplementary Material 1. [file mic-170-01493-s001.pdf]

Supplementary file

Strain	Genotype/ Description	Selectable marker	Reference
Escherichia coli			
CFT073 Wild-type	Prototypic urosepsis isolate; O6:K2:H1 serotype	None	(1, 2)
CFT073 Δ*ksl*	*ksl::kan*	Kan^R	(3)
CFT073 Δ*ksl::FRT*	*ksl::FRT*	None	This study
CFT073 Δ*waaL*	*waaL::kan*	Kan^R	This study
CFT073 Δ*waaL::FRT*	*waaL::FRT*	None	This study
CFT073Δ*ksl* Δ*waaL*	*ksl::gent, waaL::kan*	$Gent^R$, Kan^R	This study
CFT073Δ*ksl::FRT* Δ*waaL::FRT*	*ksl::FRT waaL::FRT*	None	This study
CFT073 Δ*wzy*	*wzy::kan*	Kan^R	This study
CFT073 Δ*wzy::FRT*	*wzy::FRT*	None	This study
CFT073 Δ*waaG*	*waaG::gent*	$Gent^R$	This study
CFT073 Δ*waaG::FRT*	*waaG::FRT*	None	This study
DH5α	K-12, serum sensitive control, *fhuA2 Δ(argF-lacZ)U169 phoA glnV44 Φ80 Δ(lacZ)M15 gyrA96 recA1 relA1 endA1 thi-1 hsdR17*	None	New England Biolabs

Table S1 Strains used in this study. Kan - Kanamycin, Gent – Gentamicin.

**Plasmids**	**Description**	**Resistance**	**Reference**
pCP20	Possesses FLP flip recombinase gene, 30°C temperature-sensitive replication	Amp^R	(4)
pKD4	Possesses FRT-flanked kanamycin resistance cassette	Kan^R	(5)
pMH2	Possesses gentamicin resistance cassette which was utilised in mutant construction	$Gent^R$	Hunt *et al.*, 2015, Unpublished
pKD46	Used to construct mutants through homologous recombination, possesses λ-Red recombinase genes *exo bet* and *gam* which are induced by L-arabinose, temperature sensitive replication at 30°C	Amp^R	(5)
pBWB536	Complementation of O6-antigen synthesis genes	Amp^R	(6)
pXLW36	pWKS30 with *kslCDABE* for complementation of *ksl* mutant	Amp^R	(7)
pBAD/His-WaaG	pBAD backbone; Vector used for complementation of *waaG* mutations. Under control of P*araBAD* arabinose-inducible promoter	Amp^R	(8)

Table S2 Plasmids used in this study. . Kan - Kanamycin, Gent - Gentamicin, Amp – Ampicillin.

Name	Sequence (5'-3')	Purpose
1	ATGTCGTTTTGTTGGAATGAAATTAACTCTGGTGTCAAGTCTTTAATTCTAT**TGTGTAGGCTGGAGCTGC**	**Amplify Kan^R** cassette from pKD4 with homology to *waaL*
2	TTACTTATCTAATAAACATTGGTCCGATTGTACTTTAAAATAAGCACAAAGGTC**CATATGAATATCCTCC**	**Amplify Kan^R** cassette from pKD4 with homology to *waaL*
3	GAGTCATTTGCGCACGAAAG	Screening primers for *waaL* in wild-type and mutant
4	AGATGGTTTGTAGGGCTCCG	Screening primers for *waaL* in wild-type and mutant
5	TAATGACGCAATTAAGTTATATCAAAATGATGAAAATGATGAAAATTTGAACATTTAGTATT**GTGTAGGCTGGAGCTGC**	Amplify Kan^R cassette from pKD4 with homology to *kpsT*
6	GGGTATGAATAAAGATTTTTTGTTTGGATCAAAGTCAATATCATAATTAGGTC**CATATGAATATCCTCC**	Amplify Kan^R cassette from pKD4 with homology to *kpsS*
7	GTCTTTATCAGAATATTAATGACGCAATTAAGTTATATCAAAATGATGAAAATTTGAACATTT**AGTGCGAATCCATGTGGGAGTTTA**	**Amplify Gentamicin^R** cassette from pMH2 -homology to *kpsT*
8	GAATGCATTGGGTATGAATAAAGATTTTTTGTTTGGATCAAAGTCAATATCATAATTTA**TTAGGTGGCGGTACTTGGGT**	**Amplify Gentamicin^R** cassette from pMH2 -homology to *kpsS*
9	CCCTGGTATGAAGCACGTTG	Screening primers for *ksl* operon in wild-type and mutant
10	CATGTCGTGGAGTTAAGCCG	Screening primers for *ksl* operon in wild-type and mutant
11	CGAATCCATGTGGGAGTTTA	Amplify Gentamicin^R cassette from pMH2
12	TTAGGTGGCGGTACTTGGGT	Amplify Gentamicin^R cassette from pMH2
13	TTGCCTTCCAGGCTGTTATC	*rplT* housekeeping control RT-PCR
14	CTGCTTTCGCTTTTTCAACC	*rplT* housekeeping control RT-PCR
15	CCCGTCATACTGACTGAGTACAT	*ksl2A* (region 2 capsule gene) RT-PCR
16	TGCGGTGATTTGCAGTATCC	*ksl2A* (region 2 capsule gene) RT-PCR
17	CCAGAGATTAACGCGCTCTAC	*waaQ* (R1 core biosynthesis) RT-PCR
18	TGGCACGTAATACCTTGATGAG	*waaQ* (R1 core biosynthesis) RT-PCR
19	GCTCAGCAATAGCCTCGCCGCAATTGGCGTCGACAATATA**CGAATCCATGTGGGAGTTTA**	*waaG:**gent*** mutagenesis F
20	TCGATAAATTACTTCCCTCCTCCACGACAGGTACGTCGTT**TTAGGTGGCGGTACTTGGGT**	*waaG:**gent*** mutagenesis R
21	GCAATGAAGATTGCGTTAAC	*waaG* screen F
22	AGCGTGACCGAAATGAGATG	*waaG* screen R
23	CGCAAGCGATCTGTTTACCG	*kpsC* RT-qPCR
24	TTAAGCCTGGCGCCCATAAA	*kpsC* RT-qPCR

Table S3 Oligonucleotides used in this study.

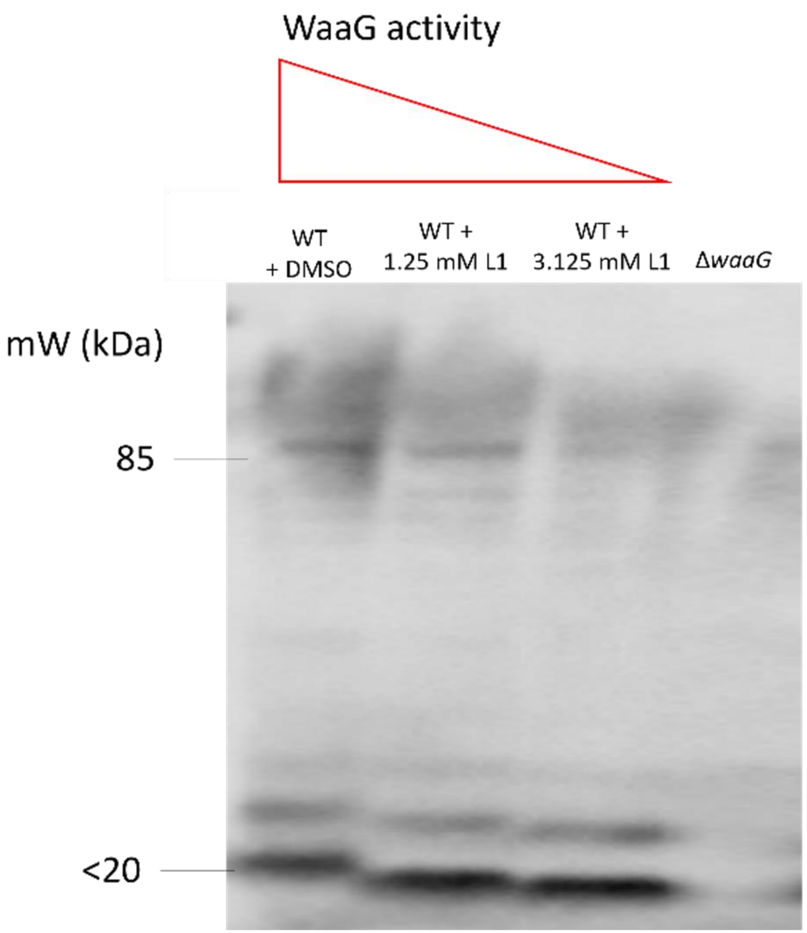

Figure S4. Dose-dependent changes to LPS in L1-treated CFT073. Whole cell lysates of the WaaG deficient mutant Δ*waaG* and Wild-type CFT073 supplemented with DMSO or L1 at concentrations of 1.25 or 3.125 mM were separated by SDS-PAGE and probed with anti-O6 antibodies for Western blot analysis of LPS and capsule. N=3 conducted, N=1 shown.

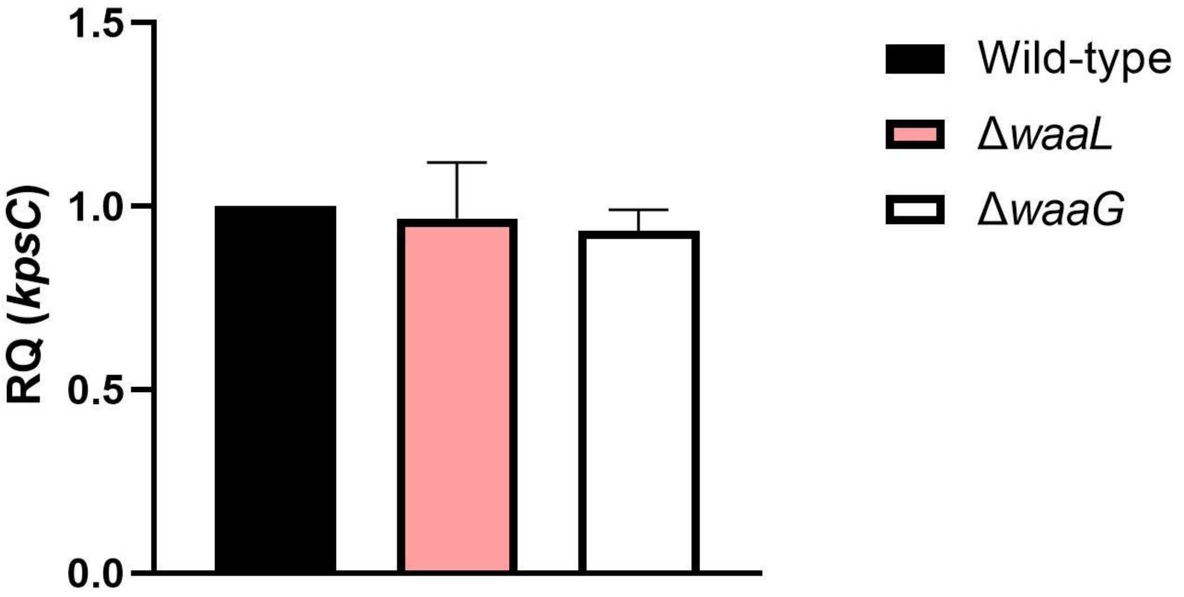

Figure S5 Region 1 *kps* gene transcription unaffected by LPS mutations. RNA was extracted from stationary phase cultures grown under standard lab conditions (LB, 37°C) and *kpsC* transcript levels were compared. Data analysis and relative expression calculation was carried out automatically on the StepOne software, with housekeeping gene *rplT* used as an internal control. Statistical analysis by One-way ANOVA and Dunnett's multiple comparisons.WT = Wild-type CFT073. N = 4.

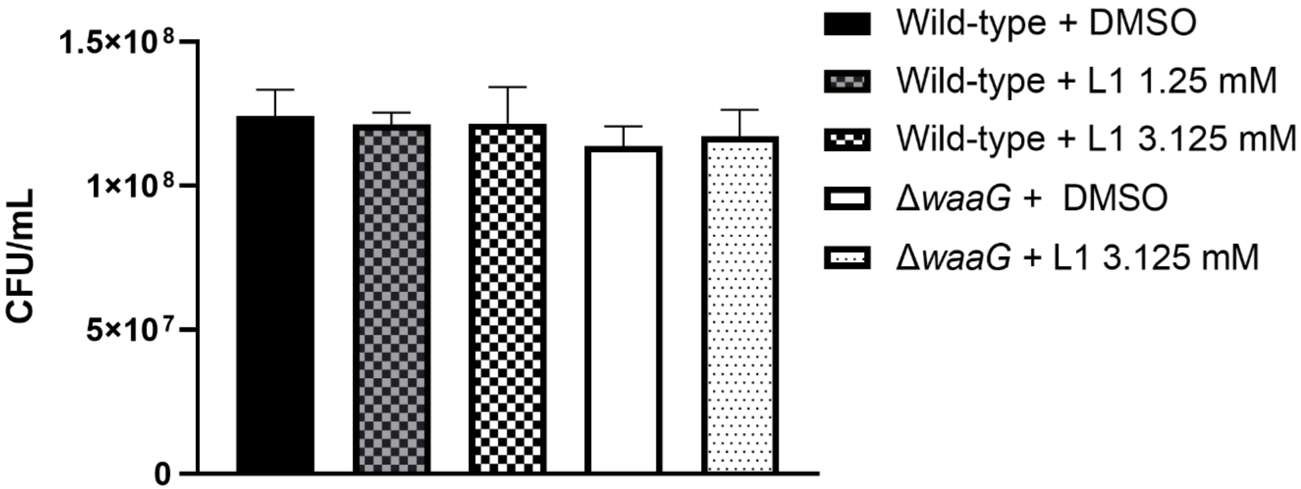

Figure S6. L1 does not display antibacterial activity towards CFT073 and mutant derivatives. Bacteria were grown overnight in LB supplemented with L1 dissolved in DMSO or DMSO. Bacteria were standardised to equivalent cell number and serially diluted prior to plating on LB agar and calculation of CFU/mL. N=3 conducted. Statistical significance was calculated by One-way ANOVA and Dunnett's multiple comparisons.

References

1. Welch RA, Burland V, Plunkett G, Redford P, Roesch P, Rasko D, et al. Extensive mosaic structure revealed by the complete genome sequence of uropathogenic Escherichia coli. Proc Natl Acad Sci U S A. 2002;99(26):17020-4.
2. Guyer DM, Kao JS, Mobley HL. Genomic analysis of a pathogenicity island in uropathogenic Escherichia coli CFT073: distribution of homologous sequences among isolates from patients with pyelonephritis, cystitis, and Catheter-associated bacteriuria and from fecal samples. Infect Immun. 1998;66(9):4411-7.
3. Miajlovic H, Cooke NM, Moran GP, Rogers TR, Smith SG. Response of extraintestinal pathogenic Escherichia coli to human serum reveals a protective role for Rcs-regulated exopolysaccharide colanic acid. Infect Immun. 2014;82(1):298-305.
4. Cherepanov PP, Wackernagel W. Gene disruption in Escherichia coli: TcR and KmR cassettes with the option of Flp-catalyzed excision of the antibiotic-resistance determinant. Gene. 1995;158(1):9-14.
5. Datsenko KA, Wanner BL. One-step inactivation of chromosomal genes in Escherichia coli K-12 using PCR products. Proc Natl Acad Sci U S A. 2000;97(12):6640-5.
6. Sarkar S, Ulett GC, Totsika M, Phan MD, Schembri MA. Role of capsule and O antigen in the virulence of uropathogenic Escherichia coli. PLoS One. 2014;9(4):e94786.
7. Buckles EL, Wang X, Lane MC, Lockatell CV, Johnson DE, Rasko DA, et al. Role of the K2 capsule in Escherichia coli urinary tract infection and serum resistance. J Infect Dis. 2009;199(11):1689-97.
8. Muheim C, Bakali A, Engström O, Wieslander Å, Daley DO, Widmalm G. Identification of a Fragment-Based Scaffold that Inhibits the Glycosyltransferase WaaG from Escherichia coli. Antibiotics (Basel). 2016;5(1).
